# Saccharin fading is not required for the acquisition of alcohol self-administration, and can alter the dynamics of cue-alcohol memory reconsolidation

**DOI:** 10.1007/s00213-017-4824-1

**Published:** 2018-02-06

**Authors:** Mickaël Puaud, Zofia Ossowska, Jordan Barnard, Amy L. Milton

**Affiliations:** Department of Psychology, University of Cambridge, Downing Site, Cambridge, CB2 3EB UK

**Keywords:** Alcohol, Drug self-administration, Memory reconsolidation, NMDA receptor antagonism, Rat

## Abstract

**Rationale:**

Animal models of alcohol-seeking are useful for understanding alcohol addiction and for treatment development, but throughput in these models is limited by the extensive pretraining required to overcome the aversive taste of ethanol. Work by Augier et al. (Psychopharmacology 231: 4561–4568, 2014) indicates that Wistar rats will self-administer alcohol without water deprivation, exposure to sweetened ethanol solutions or intermittent access to ethanol.

**Objectives and methods:**

We sought to replicate and extend the work of Augier et al. by comparing the acquisition of instrumental self-administration of ethanol in Lister-Hooded rats that had been previously saccharin faded (SF group) or not (NSF group). We also aimed to determine whether NMDA receptor antagonism with MK-801, given at memory reactivation, reduced subsequent ethanol-seeking behaviour in both groups of animals. Finally, we assessed the ethanol preference of SF and NSF rats using the two-bottle choice procedure.

**Results:**

Both SF and NSF groups acquired instrumental self-administration of ethanol, though SF rats consumed fewer of the earned reinforcers. MK-801, given at memory reactivation, had different effects on NSF and SF rats: impairing the capacity of an ethanol-paired conditioned stimulus (CS) to support reinstatement in NSF rats, and enhancing it in SF rats. Finally, neither SF nor NSF rats showed a preference for ethanol.

**Conclusions:**

Our data support those of Augier et al. (Psychopharmacology 231: 4561–4568, 2014) that pretraining is unnecessary for rats to acquire instrumental self-administration of ethanol. Indeed, saccharin fading may produce a weaker memory that extinguishes more readily, thus accounting for the different effects of MK-801 on SF and NSF rats.

**Electronic supplementary material:**

The online version of this article (10.1007/s00213-017-4824-1) contains supplementary material, which is available to authorized users.

## Introduction

Addiction to alcohol is a major societal problem, costing the European Union an estimated €62.4 billion per year (Olesen et al. 2012). Understanding the biological basis of addiction is of great utility in the development of new treatments, and animal models of addiction have been particularly useful in identifying the neurobiological substrates and psychological processes that become maladaptive during addiction. However, the study of alcohol addiction in animal models has been challenging, because establishing oral self-administration of ethanol (EtOH) solutions in animals usually requires extensive pretraining to habituate animals to the aversive taste of alcohol. Furthermore, although some procedures such as intermittent alcohol access (Carnicella et al. 2014; Simms et al. 2010; Simms et al. 2008) only involve exposure to unadulterated EtOH, other widely used procedures such as saccharin or sucrose fading (Tolliver et al. 1987) require the addition of sweet flavours to the EtOH solution to encourage consumption. This could potentially lead to confounding effects such as negative contrast between the solutions used during pretraining and those used during the acquisition of instrumental self-administration, and furthermore, sweeteners may alter the absorption and metabolism of EtOH (Roberts et al. 1999).

Recent reports (Augier et al. 2017, 2014) have demonstrated that Wistar rats will acquire instrumental self-administration of 20% EtOH without water deprivation, prior exposure to EtOH or the addition of sweet adulterants to the EtOH solution. Previous investigations (Milton et al. 2012; Schramm et al. 2016; von der Goltz et al. 2009) of the effects of disrupting the reconsolidation of EtOH-conditioned cue memories on subsequent EtOH-seeking behaviour have used the saccharin fading procedure to promote EtOH self-administration in rats. Targeting the reconsolidation of EtOH-associated cues (more formally, conditioned stimuli, or CSs) may provide a promising therapeutic strategy for the treatment of alcohol addiction. Previous work in rodent models has shown that the NMDA receptor antagonist MK-801 can disrupt the reconsolidation of the CS-EtOH memories that support the psychological processes of conditioned approach and conditioned motivation (Milton et al. 2012), and we have recently shown that the β-adrenergic receptor antagonist propranolol can be used to disrupt the reconsolidation of the memory that allows EtOH CSs to subsequently act as conditioned reinforcers (Schramm et al. 2016), so allowing those CSs to support complex chains of instrumental drug-seeking behaviour, even over long delays to reinforcement (Mackintosh 1974). Thus, the reconsolidation of the CS-EtOH memories that underlie all three ‘routes to relapse’ (Milton and Everitt 2010) can be targeted. This is supported by findings using reinstatement procedures which have greater translational relevance to alcohol addiction, in which NMDA receptor antagonists (Vengeliene et al. 2015; von der Goltz et al. 2009), propranolol (Wouda et al. 2010) or rapamycin (Barak et al. 2013), administered at memory reactivation reduced subsequent CS-induced reinstatement. Indeed, behavioural manipulations of reconsolidation have been translated to patient populations of hazardous drinkers (Das et al. 2015), illustrating how studies of the basic psychological and neurobiological mechanisms of memory reconsolidation in animals that are not ‘addicted’ to alcohol (as measured by, for instance, the ‘three criteria’ model developed for cocaine addiction; Belin et al. 2008; Deroche-Gamonet et al. 2004) can be informative for the development of treatments in clinical populations. Thus, understanding the mechanisms underlying CS-EtOH memory reconsolidation may allow for the identification of new drug targets for clinical use.

As has been noted previously (Carnicella et al. 2014), self-administration procedures are required to assess the motivational and reinforcing properties of EtOH and EtOH-associated cues. The ability to train rats on EtOH self-administration without requiring lengthy pretraining procedures such as intermittent EtOH access (Carnicella et al. 2014; Simms et al. 2010; Simms et al. 2008) or saccharin fading (Tolliver et al. 1987) would be clearly advantageous in increasing throughput in these demanding memory studies. Therefore, we sought to replicate and extend the work of Augier and colleagues (2014) in three related experiments, conducted in the same animals: first, we sought to determine whether a different strain of rat (Lister-Hooded) would acquire instrumental EtOH self-administration, without pre-exposure to EtOH or water deprivation, of a 10% EtOH reinforcer (as used previously in reconsolidation studies; Milton et al. 2012; Schramm et al. 2016). Strain differences in EtOH consumption and metabolism have been previously reported (Carnicella et al. 2014), so it cannot be assumed that all strains of rat would self-administer EtOH without prior EtOH experience. Secondly, we sought to establish whether the reconsolidation of a CS-EtOH memory in rats that had been conditioned to associate a light CS with 10% EtOH delivery in the instrumental self-administration procedure could be disrupted by the administration of the NMDA receptor antagonist MK-801. Finally, we assessed whether animals trained to self-administer 10% EtOH using this procedure showed EtOH preference as assessed through the two-bottle choice procedure (Tolliver et al. 1987), independently of their motivation to instrumentally work for EtOH, assessing any differences in both consummatory and appetitive motivation for EtOH, respectively.

## Methods

### Subjects

Subjects were 24 male Lister-Hooded rats (Charles River UK, Margate, UK), which began the experiment weighing between 257 and 307 g. The rats were housed two per cage in a vivarium under a 12-h reversed light-dark cycle (lights off at 0700).

The 12 rats that underwent the saccharin fading procedure were water restricted for the first 2 days of pretraining; they were given access to water for 2 h per day, immediately after the 1-h exposure to the saccharin solution, and had free access to food in the home cages. Thereafter, water was freely available in the home cages, and the rats were ‘mildly’ food restricted, fed in excess of 25 g/rat/day following each day’s behavioural procedures. This was in excess of the rats’ daily requirements and there was typically food left unconsumed at the end of each day. Thus, it is unlikely that rats were consuming EtOH for its caloric value. The NSF group did not undergo water restriction, but rather experienced the same regime of food restriction from 6 h prior to the start of instrumental training.

Principles of laboratory animal care were followed and all experimental procedures were conducted in accordance with both the UK Animals (Scientific Procedures) Act 1986 under Project Licence 70/7548 and EU legislation on the protection of animals used for scientific purposes (Directive 2010/63/EU).

### Behavioural apparatus

All behavioural testing, except for saccharin fading, was conducted in 12 identical lightproof and soundproof operant conditioning chambers (Med Associates Inc., St Albans, VT) equipped with two retractable levers on one side of the chamber and a liquid dispenser between them, attached to an external pump, set to deliver 0.1 ml of 10% (*v*/*v*) EtOH. The CS was a white cue light (2.5 W, 24 V) located above the active lever. The operant conditioning chambers were controlled using the Whisker Control system (Cardinal and Aitken 2010).

### Behavioural procedures

**Fig. 1:**
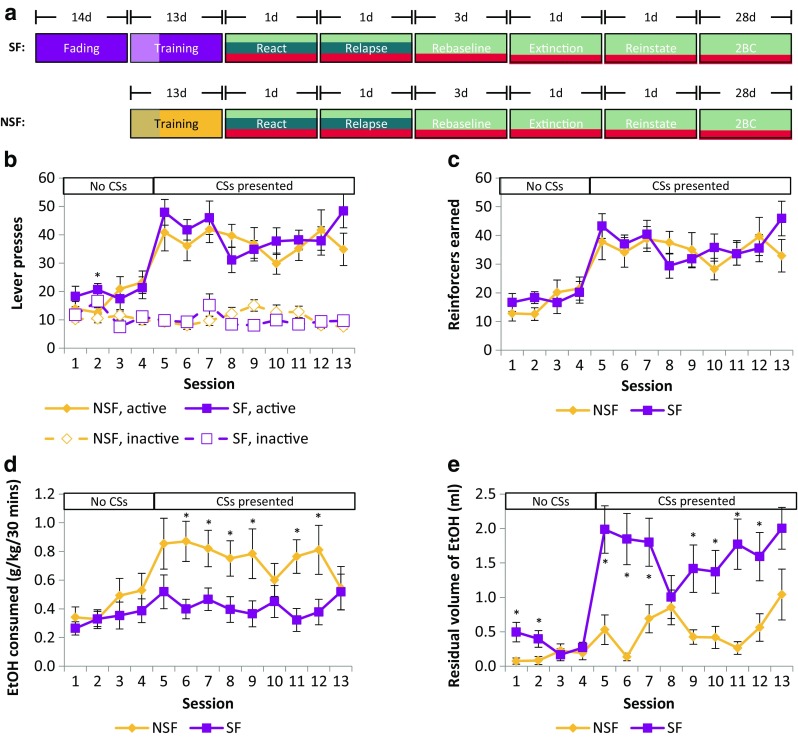
All rats acquired instrumental self-administration of 10% EtOH. Timeline of the experiments (**a**). Saccharin-faded (SF) rats underwent 14 sessions of saccharin fading prior to starting instrumental training, whereas non-saccharin-faded (NSF) rats did not. Instrumental training was split into 4 days in which the CS was not presented (indicated by the shading on the box) and 9 days when the CS was presented contingent on responding. Prior to reactivation (‘React’), rats were divided into three experimental groups, two of which received vehicle and one received the NMDAR antagonist MK-801. At test (‘Relapse’), one of the previously vehicle-treated groups did not receive the CS, while the other vehicle-treated group and the previously MK-801-treated group did. The vehicle-treated animals were pooled to form a single control group for rebaselining (‘Rebaseline’) on EtOH self-administration, extinction training and subsequent CS-induced reinstatement testing (‘Reinstate’). Finally, all animals were tested for EtOH preference and voluntary consumption using the two-bottle choice (‘2BC’) procedure. SF and NSF rats pressed the active lever, which was reinforced with 10% EtOH, more than the inactive lever, which was not reinforced (**b**). The number of 10% EtOH reinforcers earned was equivalent for both SF and NSF groups (**c**). NSF rats consumed more EtOH than SF rats (d), most likely due to consuming more of the reinforcers earned during training, as shown by lower ‘residual volumes’ of EtOH in the magazine (**e**). CSs were available from Session 5 onwards, indicated by the bars above the graphs. Group sizes: NSF, *n* = 12; SF, *n* = 12. *p < 0.05 comparison of SF to NSF rats

A full experimental timeline is shown in Fig. 1a. Twenty-four rats were divided into two equal-sized groups and trained to self-administer 10% (*v*/*v*) EtOH under two different conditions. In one condition, rats were saccharin faded before the start of instrumental EtOH self-administration training (SF group), and in the other, rats were not saccharin faded (NSF group).

#### Saccharin fading

Rats in the SF group were pretrained with 14 sessions of saccharin fading as described previously (Milton et al. 2012). Briefly, rats were transported to a holding room in individual cages and given access to a drinking bottle containing a solution with rising concentration of EtOH (Table 1) for 1 h each day. The bottles were weighed before and after each session to give a measure of fluid consumption. The subjects were water deprived for no more than 22 h per day for the first two sessions only and mildly food deprived for the remaining 12 sessions. At no point were the subjects both food and water deprived.

**Table 1:** Saccharin fading solutions (*v*/*v*) across the 14 fading sessions, as previously used in Milton et al. (2012)

Session	1–4	5–6	7–8	9–10	11–12	13–14
Solution	0.2% saccharin	0.2% saccharin + 5% EtOH	5% EtOH	0.2% saccharin + 8% EtOH	8% EtOH	0.2% saccharin + 10% EtOH

#### Instrumental EtOH self-administration training

In a procedure based upon that used by Augier et al. (2014), rats were trained in operant conditioning chambers in 30-min-long sessions over 13 days to self-administer 10% EtOH on a fixed ratio 1 (FR1) schedule, with a limit of 100 reinforcers per session. Rats were placed individually in the chambers containing one active and one inactive lever (locations counterbalanced between rats) on two sides of a drinking well where EtOH reinforcers were delivered. Presses on the active lever resulted in the delivery of a 10% EtOH solution only for sessions 1–4 and delivery of 10% EtOH solution paired with a 5-s cue light above the active lever for sessions 5–13. The omission of the cue light for the first four sessions was directly based upon the procedures used by Augier et al. (2014). Presses on the inactive lever were recorded but had no programmed consequences. Each EtOH delivery was followed by a 5-s time out period, during which time the levers were retracted. Lever presses were recorded automatically by the computer, and the amount of EtOH consumed was calculated by subtracting the volume of any EtOH remaining in the drinking well (measured directly using a 1-cm^3^ syringe) from the total volume of EtOH delivered during the session (0.1 ml per reinforcer).

#### CS-EtOH memory reactivation

The 24 rats were subsequently divided into experimental drug groups for an experiment investigating the requirement for NMDA receptor activity for the reconsolidation of the CS-EtOH memory. For the SF and NSF groups separately, animals were divided into three experimental groups (two receiving vehicle, and one receiving the NMDAR antagonist MK-801) in such a manner that their responding for EtOH and the numbers of reinforcers received during training were matched across the three groups, and across the drug and vehicle groups. Additionally, for SF animals, the groups were matched on the basis of their fluid and EtOH consumption during fading. Thirty minutes before a memory reactivation session, 10 rats received an i.p. injection of MK-801 (0.1 mg/kg, Sigma-Aldrich) and 14 rats received an i.p. injection of 0.9% physiological saline vehicle (1 ml/kg) in a location that was distinct from the room containing the conditioning chambers, and in which they had all received habituation injections of saline (1 ml/kg) on the previous day. This dose and timing of administration of MK-801 were based upon previous studies, showing that systemic injection of MK-801 can disrupt the reconsolidation of cue-fear (Lee et al. 2006b; Merlo et al. 2014) and cue-drug (Milton et al. 2008, 2012) memories.

For the memory reactivation session, which was conducted 72 h after training, rats were returned to the conditioning chamber in which they had been trained for a session in which responding on the active lever was reinforced only by presentation of the CS light, and not the delivery of 10% EtOH. This session terminated after 30 CSs had been presented, or 30 min, depending on which occurred first. The parameters of the reactivation session were chosen to induce a ‘violation of expectations’ during the reactivation session (Pedreira et al. 2004) and have been shown previously to induce the destabilisation of CS-cocaine memories (Lee et al. 2005; Milton et al. 2008). After reactivation, rats were returned to their home cages.

#### ‘Relapse’ test

Seventy-two hours after the memory reactivation session, the rats returned to the same conditioning chambers for a 30-min test session, in which responding on the active lever was reinforced by a truncated 1-s presentation of the CS light, but not 10% EtOH delivery. In a subset of the vehicle-treated animals—the VEH (no CS) group—responding on the active lever was reinforced with neither the CS nor EtOH delivery. This control group was included to provide a measure of CS control over instrumental behaviour, as has been described previously (Milton and Everitt 2012; Milton et al. 2008).

#### Rebaselining sessions

Three weeks after the relapse test, responding for 10% EtOH was re-established by returning the rats to the conditioning chambers for three ‘rebaselining’ sessions, in which active lever pressing was reinforced with the CS light and 10% EtOH, as it was during self-administration training.

#### Extinction training

The day after the last rebaselining session, rats were returned to the conditioning chambers for a single 3-h extinction session, in which responding on the active lever was reinforced with neither the CS light nor 10% EtOH. This has been shown previously to be sufficient to reduce instrumental responding for drug-associated cues (Lee et al. 2006a).

### CS-induced reinstatement test

Twenty-four hours after extinction training, the rats were returned to the conditioning chambers for a final 60-min test session. This was a within-subject test of CS-induced reinstatement; therefore, for the first 15 min of this test session, responding on the active lever was not reinforced with the CS light, and for the subsequent 45 min, responding on the active lever produced a 1-s presentation of the CS light, but no EtOH was delivered. This allowed a direct comparison of instrumental responding with and without the CS in individual subjects.

### Two-bottle choice test of EtOH preference

Following the end of instrumental training and subsequent testing, rats were housed individually and provided with intermittent 24-h (on Mondays, Wednesdays and Fridays) to two bottles containing tap water or 10% *v*/*v* EtOH for a period of 4 weeks, to assess their voluntary EtOH consumption and preference, separately from their motivation to work for EtOH. The bottles were weighed before and after each 24-h period. Solutions were prepared and changed weekly and provided at room temperature. The positions of the bottles were alternated every day to prevent side preferences. EtOH consumption (in g/kg/24 h) and EtOH preference (as a percentage of total fluid intake) were subsequently calculated. EtOH and water consumption were calculated by measuring the difference in the weight of the bottle for each rat before and after each choice session, minus the solution lost from a ‘leakage’ bottle placed in an empty cage for the same period of time.

### Statistical analysis

Data are presented as means ± s.e.m. EtOH consumption is shown per session (i.e. in 30 min) and was calculated by multiplying the amount of liquid drunk (ml) by the EtOH concentration (*v*/*v*) and the density of the liquid (0.789 g/ml), and dividing this value by the weight of the rat (kg). This formula allows for comparison of drinking behaviour across sessions and between rats, as differences in body weight are taken into account. The data were analysed using repeated measures analyses of variance (rmANOVAs) after being checked for normality, homogeneity of variance and sphericity. When sphericity assumptions were violated, the Greenhouse–Geisser correction was applied if *ε* < 0.75, and the Huynh–Feldt correction was applied if *ε* > 0.75 (Cardinal and Aitken 2006). Degrees of freedom were adjusted accordingly and are reported to 3sf. Interactions and main effects were further investigated using pairwise comparisons, which were Šidák corrected. This is a variation of the Bonferroni test that is slightly more powerful, but still produces conservative levels of *α* (Cardinal and Aitken 2006).

#### Data availability

All data accompanying this publication are available at the University of Cambridge data repository (10.17863/CAM.8697).

## Results

### Saccharin fading proceeded as observed previously in the SF group

In those rats that were saccharin faded, fading proceeded as observed previously (Milton et al. 2012), with a reduction in fluid consumption with increasing concentrations of EtOH. All SF rats consumed the solutions in the fading sessions (Supp. Fig. 1), with an overall decrease in the amount of liquid consumed as the EtOH concentration was increased [Session: *F*_(13,117)_ = 33.2, *p* < 0.001, *η*^2^ = 0.79] and no differences between the prospective experimental groups for the reconsolidation experiment [Group: *F* < 1; Session × Group: *F* < 1]. Similarly, rats consumed equivalent levels of EtOH across fading [Group: *F* < 1; Session × Group: *F*_(26,117)_ = 1.02, *p* = 0.45], with the amount consumed varying across sessions [Session: *F*_(13,117)_ = 38.3, *p* < 0.001, *η*^2^ = 0.81]. As would be expected from the varying concentration of EtOH in the solutions, Šidák-corrected pairwise comparisons revealed that Sessions 1–4 (when no EtOH was present in the solution) varied from all others [all *p*’s < 0.032]. Consumption levels were fairly stable from session 7 onwards, with some exceptions [e.g. lower levels of responding during sessions 11 and 12, when 8% EtOH without saccharin was available, compared to some other sessions].

### Both SF and NSF rats acquired 10% EtOH self-administration

All rats acquired instrumental EtOH self-administration, with both SF and NSF groups showing an increase in instrumental responding when the CS was introduced on session 5. Both groups earned similar numbers of reinforcers during training but, unexpectedly, the SF group consumed fewer of these than the NSF group.

All rats responded more on the EtOH-reinforced (active) vs the control (inactive) lever, regardless of whether they had previously undergone the saccharin fading procedure [Lever: *F*_(1,22)_ = 116, *p* < 0.001, *η*^2^ = 0.84; Lever × Pretraining: *F* < 1]. Both SF and NSF rats (Fig. 1b) increased their responding for EtOH over the course of training [Session: *F*_(9.96,219)_ = 10.6, *p* < 0.001, *η*^2^ = 0.33] with an increase in responding from session 5 onwards, when the light CS was introduced [Šidák-corrected pairwise comparisons: sessions 1–4 did not differ from each other, all *p*’s > 0.99, but differed from sessions 5–13, which also did not differ from each other, all *p*’s > 0.93]. Discrimination between the active and inactive levers improved with training, independently of any pretraining [Lever × Session: *F*_(9.81,216)_ = 20.1, *p* < 0.001, *η*^2^ = 0.48; Lever × Session × Pretraining: *F*_(9.81,216)_ = 1.25, *p* = 0.26]. Though there was a weak Session × Pretraining interaction [*F*_(9.96,219)_ = 2.08, *p* = 0.027, *η*^2^ = 0.086], this was due to higher levels of responding by rats in the SF group on a single session [session 2, SF group responded more than the NSF group, as shown by a Šidák-corrected pairwise comparison, *p* = 0.003]. Overall, there were no overall differences in the behaviour shown by the rats in the SF and NSF groups [Pretraining: *F* < 1], supporting the findings previously published by Augier et al. (2014) that rats can learn to self-administer oral EtOH without the requirement for previous saccharin fading.

As would be expected from the similarities in the acquisition of instrumental self-administration between the SF and NSF groups, there were no differences in the numbers of EtOH reinforcers (Fig. 1c) earned during training between groups [Pretraining: *F* < 1], with all animals earning greater numbers of reinforcers as training progressed [Session: *F*_(9.46,208)_ = 16.8, *p* < 0.001, *η*^2^ = 0.43; Session × Pretraining: *F*_(9.46,208)_ = 1.52, *p* = 0.14]. Interestingly, however, there were differences in the amount of EtOH consumed (in g/kg/30 min, as calculated by dividing the volume of EtOH consumed by body weight; Fig. 1d) according to the pretraining received, with rats that had previously undergone saccharin fading consuming less EtOH than rats that had not received any pretraining [Pretraining: *F*_(1,22)_ = 4.35, *p* = 0.049, *η*^2^ = 0.17; Pretraining × Session: *F*_(5.38,118)_ = 2.62, *p* = 0.024, *η*^2^ = 0.11]. Although the SF rats were heavier than, and gained weight at a slower rate than, the NSF rats (Supp. Fig. 2) due to their increased age at the start of instrumental training [Pretraining: *F*_(1,22)_ = 75.1, *p* < 0.001, *η*^2^ = 0.77; Session × Pretraining: *F*_(3.28,72.1)_ = 5.34, *p* = 0.002, *η*^2^ = 0.20], the calculation of EtOH consumption takes body weight into account, and the number of reinforcers earned in each instrumental session was far below the theoretical maximum of 360 (12 reinforcers per minute), so we do not believe that this difference reflects any sort of constraint on instrumental responding. Rather, analysis of the residual volume of EtOH left in the wells (Fig. 1e) following the end of each session indicated that the SF group, despite earning a similar number of reinforcers to the NSF group, did not consume as much of the EtOH that they had earned [Session: *F*_(5.77,127)_ = 12.6, *p* < 0.001, *η*^2^ = 0.36; Pretraining: *F*_(1,22)_ = 11.9, *p* = 0.002, *η*^2^ = 0.35; Session × Pretraining: *F*_(5.77,127)_ = 5.35, *p* < 0.001, *η*^2^ = 0.20]. This may reflect, therefore, reduced motivation to consume EtOH, but not to press levers, in the previously saccharin-faded group.

Additionally, it was verified that there were no pre-existing differences in behaviour in the prospective experimental groups for the subsequent manipulation of the CS-EtOH memory. There were no overall differences in responding for EtOH between the prospective experimental groups [Group: *F* < 1; Group × Pretraining: *F* < 1; Lever × Group: *F* < 1; Session × Group: *F* < 1; Lever × Session × Group: *F* < 1] and no differences in the numbers of reinforcers earned during training [Group: *F* < 1; Group × Pretraining: *F* < 1; Session × Group: *F* < 1; Session × Pretraining × Group: *F* < 1]. Furthermore, there were no differences in the amount of EtOH consumed by animals in the prospective drug groups [Group: *F* < 1; Group × Pretraining: *F* < 1; Session × Group: *F* < 1; Session × Group × Pretraining: *F* < 1] and no differences in the volumes of EtOH remaining in the wells at the end of the training sessions [Group: *F*_(2,18)_ = 1.01, *p* = 0.38; Group × Pretraining: *F* < 1; Session × Group: *F* < 1; Session × Pretraining × Group: *F* < 1]. For the SF rats, there were also no differences in their fluid consumption during the saccharin fading procedure [Group: *F* < 1; Session × Group: *F* < 1]. Thus, although there were differences in the training performance of SF and NSF rats, within those groups, the prospective drug groups were well matched for their EtOH exposure and responding for EtOH during training.

### All rats responded equally during a memory reactivation session

All groups responded equivalently during a brief memory reactivation session, conducted with the same parameters as training (except that active lever presses were reinforced only with the light CS and not EtOH, and the session was truncated to 30 min or 30 reinforcers, whichever occurred first). They showed similar numbers of lever presses and received the same number of CS re-exposures.

**Fig. 2:**
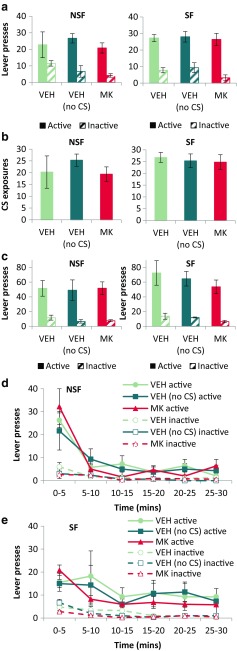
NSF and SF rats behaved similarly during memory reactivation (**a**, **b**) and test (**c**, **d**, **e**) sessions. Performance during the memory reactivation session was equivalent, regardless of pretraining or experimental drug group. Rats made similar numbers of lever presses (**a**) and received exposure to a similar number of CSs (**b**). Note that the VEH (no CS) group received exposure to the CS during reactivation. During a ‘relapse’ test, conducted 72 h later, there was no effect of either omitting the CS from the test session, or of MK-801 treatment given previously in conjunction with memory reactivation (**c**). To avoid any masking of the effect of the CS on behaviour by rapid extinction of instrumental responding during test, the test data were reanalysed in 5-min time bins for NSF (**d**) and SF (**e**) rats. NSF rats responded more during the first 5 min of the test, but there were no other differences between experimental groups. Group sizes: NSF VEH, *n* = 4; NSF VEH (no CS), *n* = 3; NSF MK, *n* = 5; SF VEH, *n* = 4; SF VEH (no CS), *n* = 3; SF MK, *n* = 5

During memory reactivation (Fig. 2a), all animals responded more on the active than the inactive lever [Lever: *F*_(1,18)_ = 66.6, *p* < 0.001, *η*^2^ = 0.79], with no differences between the SF and NSF groups [Pretraining: *F* < 1; Lever × Pretraining: *F*_(1,18)_ = 1.02, *p* = 0.33], and no differences in responding between the prospective experimental groups (those given the NMDAR antagonist MK-801 prior to memory reactivation, or its saline vehicle, and animals given saline who would subsequently have the CS omitted at test) [Group: *F*_(2,18)_ = 2.57, *p* = 0.10; Lever × Group: *F* < 1; Lever × Pretraining × Group: *F* < 1].

Similarly, there were no differences in the number of CS re-exposures (Fig. 2b) experienced by the experimental groups, as revealed by a univariate ANOVA [Group: *F* < 1; Pretraining: *F*_(1,18)_ = 1.49, *p* = 0.24; Group × Pretraining: *F* < 1]. Therefore, there were no acute effects of the MK-801 on responding or the number of CS re-exposures during the memory reactivation session.

### Responding at test was not potentiated by CS presentation

Twenty-four hours after the memory reactivation session, rats were tested in a 30-min extinction test, in which responses on the active lever were reinforced with only a truncated 1-s presentation of CS light, and not EtOH delivery (for the VEH and MK groups) or with neither the CS nor EtOH for the VEH (no CS) group. We have used similar CS omission conditions in previous experiments (Milton and Everitt 2012; Milton et al. 2008) to quantify the extent to which a pavlovian drug-associated CS can potentiate drug-seeking instrumental behaviour. However, planned comparisons of the total levels of responding in the 30-min test (Fig. 2c) revealed that, in this experiment, the VEH (no CS) group did not respond any less than the groups for which the CS was presented at test [Group: *F* < 1; Lever × Group: *F* < 1; Lever × Pretraining × Group: *F* < 1]. There was also no effect at test of NMDAR antagonism given in conjunction with memory reactivation; rather, all animals discriminated between the active and inactive levers [Lever: *F*_(1,18)_ = 115, *p* < 0.001, *η*^2^ = 0.87].

It was possible that any potentiation of the CS on instrumental responding could have been transient and masked by extinction of the CS in later stages of the test session. To investigate this possibility, the test data were reanalysed in six 5-min time bins (Fig. 2d and e). While this analysis revealed that responding, particularly on the active lever, did decrease throughout the session [Bin: *F*_(2.45,44.1)_ = 19.9, *p* < 0.001, *η*^2^ = 0.53; Lever × Bin: *F*_(4.29,77.3)_ = 10.0, *p* < 0.001, *η*^2^ = 0.36], responding did not differ on the basis of the experimental group to which rats were assigned [Group: *F* < 1; Lever × Group: *F* < 1; Bin × Group: *F* < 1; Lever × Bin × Group: *F*_(8.59,77.3)_ = 1.42, *p* = 0.20]. Therefore, consistent with the analysis of responding throughout the whole session, the CS did not potentiate responding in the VEH group vs the VEH (no CS) group, and MK-801 given at reactivation did not alter responding at subsequent test.

There were, however, some differences in responding across the test session based on whether rats had previously received pretraining with saccharin. NSF rats responded more on the active lever at the beginning of the session [Lever × Bin × Pretraining: *F*_(4.29,77.3)_ = 4.16, *p* = 0.003, *η*^2^ = 0.19; Šidák-corrected pairwise comparisons revealed that responding on the active lever differed between the NSF and SF groups for the first 5 min of testing (*p* = 0.044), but not for subsequent bins (all *p*’s > 0.05), and there were no differences in responding on the inactive lever (all *p*’s > 0.20)].

Overall, however, it would appear that the EtOH-associated CS did not potentiate responding during an extinction test. The unexpected lack of effect of CS omission in the control group indicates that responding at test was *not* influenced by the presence of the pavlovian CS from training, contrary to our previous observations in similar, though not identical, procedures (Milton and Everitt 2012; Milton et al. 2008). This is also in spite of a clear increase of responding during training following the introduction of the CS (see above). We would speculate, therefore, that the difference between our previous and current observations is due to the later introduction of the EtOH-associated CS during training (session 5 in the current study; throughout training in our Milton and Everitt 2012 and Milton et al. 2008). This may have produced an instrumental lever press-EtOH memory that was relatively stronger than the pavlovian memory in the current study as compared to our previous work. This was assessed in the next stage of the experiment, where animals were first rebaselined on EtOH self-administration and subsequently underwent the ‘extinction-reinstatement’ procedure (Davis and Smith 1976).

### All groups responded equivalently during rebaselining sessions

Following the conclusion of the reconsolidation experiment, rats were returned to the conditioning chambers for three rebaselining sessions to re-establish instrumental responding for EtOH (Supp. Fig. 3). As the VEH and VEH (no CS) groups had received the same drug treatment prior to memory reactivation, these animals were pooled to create a single VEH control group for the rest of the experiment.

All animals, regardless of whether they had undergone saccharin fading or not, and irrespective of the drug treatment given prior to memory reactivation, reacquired instrumental responding for EtOH. There were no overall differences in the number of EtOH reinforcers earned, and in contrast to EtOH consumption during the acquisition of instrumental responding for EtOH, there were no differences in consumption between SF and NSF rats. However, there was reduced consumption of EtOH specifically in the SF group that had received MK prior to memory reactivation.

All rats reacquired responding for EtOH in the rebaselining sessions, rapidly reacquiring responding on the active lever [Lever: *F*_(1,20)_ = 107, *p* < 0.001, *η*^2^ = 0.84]. There were no overall differences in responding (Supp. Fig. 3a, b) between the SF and NSF rats [Pretraining: *F*_(1,20)_ = 2.50, *p* = 0.13] or between experimental groups that were administered different drugs prior to memory reactivation [Drug: *F* < 1] and no interaction [Pretraining × Drug: *F*_(2,20)_ = 0.13]. There were no differences in the numbers of CS and EtOH reinforcers earned (Supp. Fig. 3c, d) during the rebaselining sessions [Pretraining: *F*_(1,20)_ = 4.18, *p* = 0.054; Drug: *F* < 1; Pretraining × Drug: *F*_(1,20)_ = 2.81, *p* = 0.11], though there was a difference in EtOH consumption (Supp. Fig. 3e, f) in grams per kilogram per 30 min depending on both the pretraining history of the animal and the drug treatment that it had previously received [Pretraining × Drug: *F*_(1,20)_ = 5.90, *p* = 0.025, *η*^2^ = 0.23; Pretraining: *F*_(1,20)_ = 2.57, *p* = 0.12; Drug: *F*_(1,20)_ = 1.80, *p* = 0.20]. Šidák-corrected pairwise comparisons showed that this was due to low levels of EtOH consumption in the SF MK group [orthogonal comparisons showed no differences in EtOH consumption in the NSF groups (*p* = 0.45) or VEH groups (*p* = 0.53), but lower levels of consumption in the SF MK group as compared to the NSF MK group (*p* = 0.016) and the SF VEH group (*p* = 0.015)]. Finally, in contrast to the initial training, there were no differences in the amount of EtOH remaining in the wells (Supp. Fig. 3g, h) at the end of the rebaselining sessions [Pretraining: *F* < 1; Group: *F* < 1; Pretraining × Group: *F* < 1].

### All groups extinguished instrumental responding in the absence of the CS

All rats extinguished instrumental responding in a single 3-h session (Supp. Fig. 4) in which neither the CS nor the 10% EtOH US was presented. All rats reduced their overall levels of responding over the 3-h session, which was analysed in 5-min time bins [Bin: *F*_(35,700)_ = 22.3, *p* < 0.001, *η*^2^ = 0.53]. The reductions in responding were greatest for the active lever, which had previously been reinforced with 10% EtOH [Lever × Bin: *F*_(35,700)_ = 12.6, *p* < 0.001, *η*^2^ = 0.39]. There were no overall differences in extinction between those rats that had been saccharin faded and those that had not, no differences between the experimental groups previously given different drugs prior to the memory reactivation session and no interaction [Pretraining: *F*_(1,20)_ = 2.80, *p* = 0.11; Drug: *F* < 1; Pretraining × Drug: *F* < 1]. Although extinction lever pressing differed depending on both the pretraining and drug history of the rat [Lever × Bin × Pretraining × Drug: *F*_(35,700)_ = 2.21, *p* < 0.001, *η*^2^ = 0.10], Šidák-corrected pairwise comparisons revealed that this was primarily due to a high level of responding in the first 5 min by rats in the SF MK group [*p* = 0.04 as compared to the SF VEH group, *p* = 0.015 as compared to the NSF MK group]. Importantly, by the end of extinction training, there were no differences between animals that had been saccharin faded or not [for active lever presses in the last hour of extinction training, all *p*’s > 0.10 for NSF vs. SF groups] and those that had received VEH or MK [for active lever presses in the last hour of extinction training, all *p*’s > 0.16 for VEH vs. MK groups].

### Performance on a CS-induced reinstatement test depended upon prior saccharin exposure and the drug treatment administered during memory reactivation

**Fig. 3:**
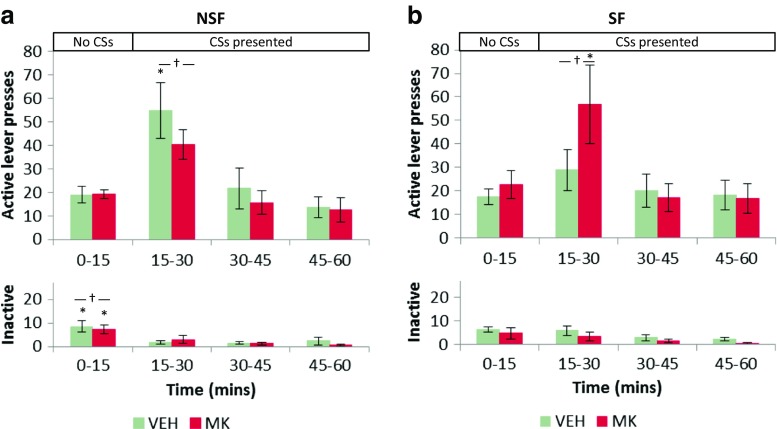
Performance in a second CS-induced reinstatement test, conducted after instrumental extinction, revealed differences in behaviour dependent upon the pretraining history of the animal and treatment at a prior memory reactivation session. NSF animals that had been treated with saline vehicle at memory reactivation increased responding on the active lever when the CS was re-introduced 15 min into the test session, while animals that had received MK-801 did not (**a**). For the SF rats, only animals that had received MK-801 showed a reliable CS-induced reinstatement of responding (**b**). Group sizes: NSF VEH, *n* = 7; NSF MK, *n* = 5; SF VEH, *n* = 7; SF MK, *n* = 5. †*p* < 0.05 compared to pressing on the same lever for other time bins in the session. *p < 0.05 compared to pressing on the same lever by the same drug group for other time bins in the session

All rats biased their responding to the active lever during the test session (Fig. 3) [Lever: *F*_(1,20)_ = 47.4, *p* < 0.001, *η*^2^ = 0.70], regardless of whether they had been previously saccharin faded, or whether they had previously received MK-801 at reactivation [Lever × Pretraining: *F* < 1; Lever × Drug: *F* < 1; Lever × Pretraining × Drug: *F*_(1,20)_ = 3.11, *p* = 0.093]. Responding differed across the course of the test session [Bin: *F*_(2.53, 50.5)_ = 15.8, *p* < 0.001, *η*^2^ = 0.44], with responding between 15 and 30 min, when the CS was first reintroduced, differing from the rest of the test session [Šidák-corrected pairwise comparisons revealed that this 15-min time bin differed from all others (all *p*’s < 0.03), which did not differ from each other (all *p*’s > 0.066)]. This change in responding was selective to the active lever, which increased when the CS was first reintroduced [Lever × Bin: *F*_(3,60)_ = 18.6, *p* < 0.001, *η*^2^ = 0.48; Šidák-corrected pairwise comparisons revealed that responding on the active lever between 15 and 30 min differed from all other time bins (all *p*’s < 0.004), which did not differ from each other (all *p*’s > 0.65). Inactive lever pressing was also higher for the first 15 min of the session, in the absence of the CS (all *p*’s < 0.041) than for the rest of the session (all *p*’s > 0.25).]

Responding during the test session was influenced by both the pretraining history of the animal and the treatment that had been administered at memory reactivation [Bin × Pretraining × Drug: *F*_(2.52,50.5)_ = 3.88, *p* < 0.02, *η*^2^ = 0.16; Lever × Bin × Pretraining × Drug: *F*_(3,60)_ = 6.44, *p* ≤ 0.001, *η*^2^ = 0.24]. Specifically, non-saccharin-faded rats (Fig. 3a) that received vehicle at reactivation showed increased responding at test when the CS was reintroduced, which subsequently reduced to baseline levels for the rest of the session [Šidák-corrected pairwise comparisons revealed that responding between 15 and 30 min differed from the rest of the time bins in the test session (*p* = 0.096 compared to 0–15 min, and *p*’s < 0.017 for the rest of the session), which did not differ from each other (all *p*’s > 0.41)]. There were no differences in responding throughout the test session for NSF rats that had received MK-801 at reactivation [all *p*’s > 0.40]. For the saccharin-faded rats (Fig. 3b), there were no differences in responding across the session in the VEH group [all *p*’s > 0.54], but rats that had received MK at reactivation showed greater responding when the CS was first reintroduced as compared to later in the test session [*p* = 0.12 compared to the first 0–15 min of the session, but all *p*’s < 0.011 compared to the rest of the session].

Further analysis of responding on the individual levers revealed that these differences in responding were selective to the active lever; previously vehicle-treated NSF animals responded more on the active lever for the first 15 min after the CS was reintroduced [all *p*’s < 0.012], with no other differences in the test session [all *p*’s > 0.53], while inactive lever pressing only differed in these animals for the first 15 min of the test [all *p*’s < 0.019 as compared to the rest of the session, which did not differ from each other, all *p*’s > 0.98]. CS presentation did not increase active lever pressing in NSF rats that had been previously treated with MK-801 [all *p*’s > 0.52], though inactive lever pressing decreased between the first 15 min and the last 15 min of the session [*p* = 0.034].

For previously saccharin-faded rats (Fig. 3b), a different pattern of responding was observed during the second test. For SF animals, rats that had received vehicle during memory reactivation did not alter their responding across the second test; neither on the active lever [all *p*’s > 0.71] nor the inactive lever [all *p*’s > 0.18]. However, rats that had received MK-801 at reactivation increased responding on the active lever when the CS was reintroduced [responding was higher during 15–30 min than 30–60 min (all *p*’s < 0.008) and trended towards being greater than the first 15 min (*p* = 0.057)] with no differences in inactive lever pressing across the test session [all *p*’s > 0.25].

In summary, SF and NSF rats responded differently during the second test session, depending on their previous history of drug treatment at memory reactivation. For NSF rats, rats that had previously received vehicle at memory reactivation showed a CS-induced reinstatement of responding when the CS was reintroduced 15 min into the test session. By contrast, rats that had previously received MK-801 at reactivation did not reinstate their responding on the active lever when the CS was reintroduced. For SF rats, a different pattern of responding was observed; for these animals, *only* those that had received MK-801 at memory reactivation reinstated their active lever responding when the CS was reintroduced.

### Neither the SF nor the NSF rats showed preference for EtOH over water

**Fig. 4:**
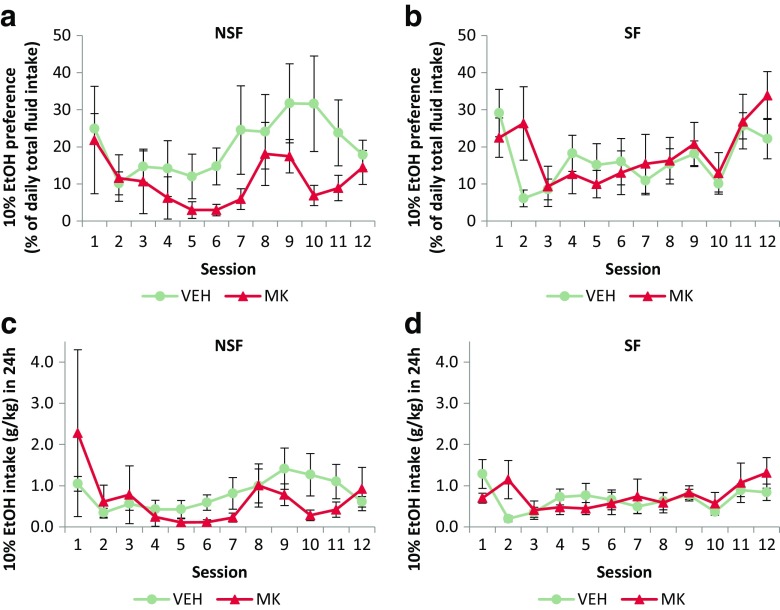
There were no differences in preference for 10% EtOH (**a**, **b**) or intake of 10% EtOH (**c**, **d**) over 24 h as assessed by the two-bottle preference test. Group sizes: NSF VEH, *n* = 7; NSF MK, *n* = 5; SF VEH, *n* = 7; SF MK, *n* = 5

Pretraining did not affect the levels of EtOH preference (Fig. 4a, b) shown by the rats, both of which equally preferred to drink water over 10% EtOH when given the opportunity in the two-bottle preference test [Pretraining: *F* < 1; Pretraining × Session: *F*_(4.42,88.4)_ = 1.68, *p* = 0.16]. Previous administration of MK-801 or vehicle during the CS-EtOH memory reactivation session also had no effect on subsequent EtOH preference in the two-bottle choice procedure [Drug: *F* < 1; Drug × Session: *F*_(4.42,88.4)_ = 1.45, *p* = 0.22] and this was the case for both NSF and SF rats [Pretraining × Drug: *F*_(1,20)_ = 1.54, *p* = 0.23; Pretraining × Drug × Session: *F* < 1]. The amount of EtOH consumed in the 24-h test sessions (Fig. 4c, d) also did not differ between the NSF and SF groups [Pretraining: *F* < 1; Pretraining × Session: *F*_(2.38,47.7)_ = 1.18, *p* = 0.32] or VEH- and MK-treated rats [Drug: *F* < 1; Drug × Session: *F*_(2.38,47.7)_ = 1.08, *p* = 0.36] and there were no differences in consumption based on the combination of their pretraining and drug history [Pretraining × Drug: *F* < 1; Pretraining × Drug × Session: *F*_(2.38,47.7)_ = 1.50, *p* = 0.23]. These data indicate that although the animals acquired instrumental responding reinforced by a 10% EtOH reinforcer, they did not show a preference for EtOH over water and titrated their EtOH intake to relatively low levels.

## Discussion

It has recently been reported by Augier et al. (2017, 2014) that Wistar rats will acquire instrumental self-administration of 20% ethanol (EtOH) without pretraining of any sort, sucrose/saccharin fading (Tolliver et al. 1987) or extensive intermittent EtOH access (Carnicella et al. 2014; Simms et al. 2010; Simms et al. 2008). Here, we sought to replicate and extend their work in three ways: (i) by using a different strain of rat and a different concentration of EtOH reinforcer; (ii) by assessing the subsequent preference of these animals for 10% EtOH over water using the two-bottle choice procedure, allowing their voluntary free consumption of EtOH to be assessed independently of their motivation to *work* for EtOH; and, (iii) by assessing the sensitivity of the CS-EtOH memories trained on this procedure to antagonism at NMDA receptors during memory reactivation. Consistent with the findings of Augier et al. (2014), we found that prior saccharin fading did not affect the capacity of Lister-Hooded rats to acquire instrumental responding for a 10% EtOH reinforcer. However, although we did not observe differences in the amount of 10% EtOH *earned* by the SF and NSF rats, we did observe differences in the amount of EtOH *consumed*, with the SF group drinking fewer of the earned reinforcers than the NSF group; some potential explanations for this are considered presently. Despite these differences in consumption during the instrumental self-administration sessions, there were no differences in consumption of, or preference for, 10% EtOH as measured by the two-bottle choice procedure; all rats titrated their intake to relatively low levels, and neither group showed a preference for 10% EtOH over water. This is in contrast to the levels of EtOH preference observed with other, more extensive pretraining procedures, such as intermittent access to two-bottle choice (Carnicella et al. 2014).

Also consistent with the findings of Augier et al. (2014), we found that the introduction during training of a pavlovian conditioned stimulus (CS) paired with reinforcer delivery increased responding selectively on the lever paired with EtOH delivery. However, although the introduction of the CS increased responding reliably in SF and NSF rats—as compared to prior sessions in which the CS was not presented—the EtOH-associated CS did not potentiate responding in a subsequent test of CS-induced reinstatement. This test, conducted 72 h after a brief memory reactivation session in which rats were treated with either the NMDA receptor antagonist MK-801 or its vehicle, showed that omission of the CS in a subset of the vehicle-treated animals did not reduce levels of active lever pressing. This contrasts with data reported previously (Milton and Everitt 2012; Milton et al. 2008) where animals that had been trained with a pavlovian CS, paired with drug and produced by lever pressing from the start of training, showed reduced active lever pressing if the CS was omitted at subsequent test. One potential explanation for this apparent discrepancy is the use of EtOH as a reinforcer in this study, as our previous experiments (Milton and Everitt 2012; Milton et al. 2008) used cocaine; however, this seems unlikely, as reinstatement induced by EtOH-associated CSs has been previously reported (Vengeliene et al. 2015; von der Goltz et al. 2009; Wouda et al. 2010). We suggest that the reduction in the effects of the CS on reinstatement behaviour likely reflects the reduced contingency of CS and EtOH pairing, as in the present experiments EtOH was presented without the CS for the first 4 days of training, in accordance with one of our aims, to replicate the findings of Augier et al. (2014). This may have shifted the balance in the control of responding towards the more predictive instrumental association and relatively weakened the contribution of the pavlovian CS; namely, the Pavlovian CS-EtOH may have been overshadowed by the instrumental association (Garrud et al. 1981). Thus, responding during the reinstatement test may have been dominated by the instrumental association, with the weaker pavlovian CS failing to potentiate responding from a relatively high instrumental baseline.

We also aimed to assess the effects of disrupting CS-EtOH memory reconsolidation by administering the NMDA receptor antagonist MK-801 or its vehicle prior to the brief memory reactivation session, in which instrumental responding was reinforced only with the CS light. In contrast to previous reports, MK-801 did not reduce responding for the CS alone at subsequent test; however, as the behaviour of the CS-omission group unexpectedly did not differ from vehicle-treated animals in which the CS was presented, the apparent lack of effect of MK-801 in this experiment is complicated to interpret. One possibility is that any effect of MK-801 on the reconsolidation of the pavlovian CS-EtOH memory was masked by expression of the instrumental memory associating active lever pressing with EtOH delivery, similar to the explanation for the lack of CS potentiation given above. MK-801 had no effect on the reconsolidation of the instrumental memory; however, although it is possible to disrupt instrumental memories with MK-801 (Exton-McGuinness and Lee 2015; Exton-McGuinness et al. 2014), this requires a different type of reactivation procedure than that used in these experiments and in previous work (Brown et al. 2008; Milton et al. 2008)—namely, a shift in reinforcement contingency at reactivation—and so, it is not surprising that there was preserved discrimination between the active and inactive levers at test. Therefore, the intact instrumental memory may have masked an impaired pavlovian memory in the MK-801 group, which was not sufficiently strong to influence overall levels of behaviour at the first CS-induced reinstatement test (hence, the lack of effect of CS omission in the control group).

In order to investigate this possibility, responding for 10% EtOH was rebaselined, and then the amount of responding supported by the instrumental memory was reduced by instrumental extinction training in the absence of the CS. Animals were subsequently retested on CS-induced reinstatement; this time using a within-subjects design to increase power, in which animals responded for the first 15 min of the test session in the absence of the CS, and subsequently with CS presentations on pressing of the active lever. With the reduced baseline of instrumental responding, CS-induced reinstatement was observed at this test; however, this manipulation revealed the surprising result that pretraining and treatment history affected CS-induced reinstatement. For NSF rats, animals that had received vehicle injections at memory reactivation showed a robust CS-induced reinstatement effect, while animals that had received MK-801 at reactivation did not. By contrast, for SF rats, *only* animals that had received MK-801 at memory reactivation showed a robust CS-induced reinstatement effect.

It therefore appears that saccharin fading influenced not only the rewarding properties of the 10% EtOH reinforcer—as the SF rats did not consume as many of EtOH reinforcers that they had earned—but also the capacity of the CS-EtOH memory to destabilise at reactivation. We speculate that, in the NSF group, the pavlovian CS-drug memory destabilised during reactivation, and its restabilisation was blocked by NMDA receptor antagonism. However, the effects of this disruption were not observed at the first test, because the strong instrumental memory masked the effects of the pavlovian CS—as supported by equivalent levels of responding in the CS-omission control group. Both vehicle-treated and MK-801-treated animals responded equivalently on the rebaselining sessions, but it has previously been shown that disruption of a pavlovian memory prevents new learning about the same CS (Lee et al. 2006a). Following extinction of the instrumental memory, the second CS-induced reinstatement test revealed the CS-EtOH memory deficit that had previously been induced by NMDA receptor antagonism at reactivation.

In the SF group, we speculate that prior saccharin fading reduced the strength of the association of the CS with the 10% EtOH as a reinforcer through negative contrast (Crespi 1942), by which animals respond less for a reinforcer when they have previously been reinforced with one of greater magnitude. We suggest that the contrast between the 10% EtOH adulterated with saccharin at the end of the fading procedure and the 10% EtOH delivered in the self-administration sessions led the SF group to consume fewer of the reinforcers earned, so producing the higher residual volumes of EtOH in the receptacle at the end of the training sessions. Although the interpretation of these data is potentially complicated by differences in the weight of the animals, it is notable that the SF group—which were heavier than the NSF group (Supp Fig. 2)—consumed *less* EtOH, so differences in consumption cannot be attributable to animals attempting to titrate their intake to a specific blood ethanol level. Furthermore, because there were no differences in the free consumption of EtOH as measured in the two-bottle choice procedure, the lower consumption of EtOH by SF animals during instrumental training appears to be transient. This is most consistent with a negative contrast interpretation, which has been shown to dissipate with time (Pellegrini et al. 2004). This finding, along with concerns that sweeteners may reduce blood EtOH levels for a particular volume of EtOH consumed (Roberts et al. 1999), would suggest that saccharin/sucrose fading is a far from optimal method for EtOH pre-exposure.

Interestingly, this negative contrast did *not* affect the motivation of the animals to work for the EtOH reinforcer, suggesting a dissociation between appetitive and consummatory behaviour in this procedure. However, the reduction in reinforcer value may have led to the formation of a weaker CS-EtOH memory in the SF group as compared to the NSF group. It has been extensively documented for fear memories that the parameters of memory reconsolidation vs extinction differ depending on the strength of the memory (Inda et al. 2011; Reichelt and Lee 2012). Thus, in the SF group, the brief memory reactivation session—which is procedurally an extinction session—may have shifted the balance from reconsolidation to extinction, and MK-801 has been widely documented as causing deficits in the consolidation of extinction memory (Baker and Azorlosa 1996; Lee et al. 2006b; Port and Seybold 1998). Thus, in the SF group, we suggest that MK-801 blocked extinction of the weak CS-EtOH memory, appearing to paradoxically strengthen it. This was only revealed at the subsequent CS-induced reinstatement test, where the weak CS-EtOH memory was not capable of supporting CS-induced reinstatement in the vehicle-treated group, but could in animals that had previously received MK-801.

It should be noted that although saccharin fading may have altered the dynamics of CS-EtOH memory reconsolidation in this study, this is not an intrinsic characteristic of saccharin fading in itself, but rather likely reflects the specific combination of pretraining and training procedures used. Saccharin fading has been used previously in studies of memory reconsolidation, with successful destabilisation of the CS-EtOH memory (Milton et al. 2012; Schramm et al. 2016; von der Goltz et al. 2009). However, in these studies, the pavlovian CSs were available from the start of EtOH self-administration training, so overshadowing of the pavlovian memory by the instrumental association is unlikely (Garrud et al. 1981). Indeed, in the studies of conditioned reinforcement conducted by Schramm et al. (2016), there cannot be overshadowing of the pavlovian memory by the instrumental response in the test phase, because these studies assessed the integrity of the CS-EtOH memory after reconsolidation through the impact of the CS, acting as a conditioned reinforcer, to support the acquisition of a new instrumental-seeking response; this is a stringent test of the CS-EtOH association (Mackintosh 1974). Therefore, in the work described here, it is likely that the reduced value of the EtOH (due to negative contrast in the SF group), in the context of a pavlovian CS-EtOH memory that was partially overshadowed by an instrumental memory, led to a sufficiently weak CS-EtOH memory that the re-exposure biased towards extinction of the memory, rather than its reactivation. We speculate that a shorter memory reactivation session in the SF group would have also led to memory destabilisation and sensitivity to MK-801, but a parametric analysis of this was beyond the scope of the current work.

In conclusion, our data support the assertion of Augier et al. (2014) that pretraining exposure to EtOH is not required for the acquisition of instrumental EtOH self-administration, in Lister-Hooded as well as Wistar rats. Furthermore, our data extend the previous work by showing that animals will work for an EtOH reinforcer that they only voluntarily consume in small amounts and that they do not prefer over water. Thus, while useful in respect of the shortened training, the Augier procedure does not appear to support levels of EtOH consumption as high as other, more extended pretraining procedures, such as intermittent access (Carnicella et al. 2014). However, the Augier procedure does appear to produce a stronger CS-EtOH memory than procedures involving saccharin fading, at least when the EtOH-associated CS is not present throughout self-administration training. Overall, we would suggest that saccharin fading is not an optimal pretraining procedure for EtOH studies, and that the Augier procedure represents an improvement to this. However, in light of our data showing that neither the NSF nor the SF animals in these experiments preferred EtOH over water when given a free choice, we would recommend the use of pretraining procedures that do increase this, such as the intermittent alcohol access to two-bottle choice procedures originally developed by Wise (1973) and further investigated by Ron and colleagues (Carnicella et al. 2014). Our data also support previous findings that NMDA receptor antagonism during the reactivation of a CS-drug memory can reduce subsequent CS-induced reinstatement by extending this to a CS-EtOH memory.

## Electronic supplementary material

ESM 1 (PDF 328 kb)

## References

[CR1] Augier E, Flanigan M, Dulman RS, Pincus A, Schank JR, Rice KC, Kejun C, Heilig M, Tapocik JD (2014). Wistar rats acquire and maintain self-administration of 20% ethanol without water deprivation, saccharin/sucrose fading, or extended access training. Psychopharmacology.

[CR2] Augier E, Dulman RS, Singley E, Heilig M (2017) A method for evaluating the reinforcing properties of ethanol in rats without water deprivation, saccharin fading or extended access training. J Vis Exp 119: doi: 10.3791/53305 10.3791/53305 PMC5352301 28190044

[CR3] Baker JD, Azorlosa JL (1996). The NMDA antagonist MK-801 blocks the extinction of Pavlovian fear conditioning. Behav Neurosci.

[CR4] Barak S, Liu F, Ben Hamida S, Yowell QV, Neasta J, Kharazia V, Janak PH, Ron D (2013). Disruption of alcohol-related memories by mTORC1 inhibition prevents relapse. Nat Neurosci.

[CR5] Belin D, Mar AC, Dalley JW, Robbins TW, Everitt BJ (2008). High impulsivity predicts the switch to compulsive cocaine-taking. Science.

[CR6] Brown TE, Lee BR, Sorg BA (2008). The NMDA antagonist MK-801 disrupts reconsolidation of a cocaine-associated memory for conditioned place preference but not for self-administration in rats. Learn Mem.

[CR7] Cardinal RN, Aitken MRF (2006) ANOVA for the behavioural sciences researcher. Lawrence Erlbaum Associates, Inc

[CR8] Cardinal RN, Aitken MRF (2010). Whisker: a client-server high-performance multimedia research control system. Behav Res Methods.

[CR9] Carnicella S, Ron D, Barak S (2014). Intermittent ethanol access schedule in rats as a preclinical model of alcohol abuse. Alcohol.

[CR10] Crespi LP (1942). Quantitative variation of incentive and performance in the white rat. Am J Psychol.

[CR11] Das RK, Lawn W, Kamboj SK (2015). Rewriting the valuation and salience of alcohol-related stimuli via memory reconsolidation. Transl Psychiatry.

[CR12] Davis WM, Smith SG (1976). Role of conditioned reinforcers in the initiation, maintenance and extinction of drug-seeking behavior. Pavlov J Biol Sci.

[CR13] Deroche-Gamonet V, Belin D, Piazza PV (2004). Evidence for addiction-like behavior in the rat. Science.

[CR14] Exton-McGuinness MTJ, Lee JLC (2015). Reduction in responding for sucrose and cocaine reinforcement by disruption of memory reconsolidation. eNeuro.

[CR15] Exton-McGuinness MTJ, Patton RC, Sacco LB, Lee JLC (2014). Reconsolidation of a well-learned instrumental memory. Learn Mem.

[CR16] Garrud P, Goodall G, Mackintosh NJ (1981). Overshadowing of a stimulus-reinforcer association by an instrumental response. Q J Exp Psychol B.

[CR17] von der Goltz C, Vengeliene V, Bilbao A, Perreau-Lenz S, Pawlak CR, Kiefer F, Spanagel R (2009). Cue-induced alcohol seeking behaviour is reduced by disrupting the reconsolidation of alcohol-related memories. Psychopharmacology.

[CR18] Inda MC, Muravieva EV, Alberini CM (2011). Memory retrieval and the passage of time: from reconsolidation and strengthening to extinction. J Neurosci.

[CR19] Lee JLC, Di Ciano P, Thomas KL, Everitt BJ (2005). Disrupting reconsolidation of drug memories reduces cocaine seeking behavior. Neuron.

[CR20] Lee JLC, Milton AL, Everitt BJ (2006). Cue-induced cocaine seeking and relapse are reduced by disruption of drug memory reconsolidation. J Neurosci.

[CR21] Lee JLC, Milton AL, Everitt BJ (2006). Reconsolidation and extinction of conditioned fear: inhibition and potentiation. J Neurosci.

[CR22] Mackintosh NJ (1974) The psychology of animal learning. Academic Press

[CR23] Merlo E, Milton AL, Goozée ZY, Theobald DEH, Everitt BJ (2014). Reconsolidation and extinction are dissociable and mutually exclusive processes: behavioral and molecular evidence. J Neurosci.

[CR24] Milton AL, Everitt BJ (2010). The psychological and neurochemical mechanisms of drug memory reconsolidation: implications for the treatment of addiction. Eur J Neurosci.

[CR25] Milton AL, Everitt BJ (2012). The persistence of maladaptive memory: addiction, drug memories and anti-relapse treatments. Neurosci Biobehav Rev.

[CR26] Milton AL, Lee JLC, Butler VJ, Gardner RJ, Everitt BJ (2008). Intra-amygdala and systemic antagonism of NMDA receptors prevents the reconsolidation of drug-associated memory and impairs subsequently both novel and previously acquired drug-seeking behaviors. J Neurosci.

[CR27] Milton AL, Schramm MJW, Wawrzynski J, Gore F, Oikonomou-Mpegeti F, Wang NQ, Samuel D, Economidou D, Everitt BJ (2012). Antagonism at NMDA receptors, but not β-adrenergic receptors, disrupts the reconsolidation of Pavlovian conditioned approach and instrumental transfer for ethanol-associated conditioned stimuli. Psychopharmacology.

[CR28] Olesen J, Gustavsson A, Svensson M, Wittchen HU, Jönsson B, group Cs, Council EB (2012). The economic cost of brain disorders in Europe. Eur J Neurol.

[CR29] Pedreira ME, Pérez-Cuesta LM, Maldonado H (2004). Mismatch between what is expected and what actually occurs triggers memory reconsolidation or extinction. Learn Mem.

[CR30] Pellegrini S, Muzio RN, Mustaca AE, Papini MR (2004). Successive negative contrast after partial reinforcement in the consummatory behavior of rats. Learn Motiv.

[CR31] Port RL, Seybold KS (1998). Manipulation of NMDA-receptor activity alters extinction of an instrumental response in rats. Physiol Behav.

[CR32] Reichelt AC, Lee JLC (2012). Appetitive Pavlovian goal-tracking memories reconsolidate only under specific conditions. Learn Mem.

[CR33] Roberts AJ, Heyser CJ, Koob GF (1999). Operant self-administration of sweetened versus unsweetened ethanol: effects on blood alcohol levels. Alcohol Clin Exp Res.

[CR34] Schramm MJW, Everitt BJ, Milton AL (2016). Bidirectional modulation of alcohol-associated memory reconsolidation through manipulation of adrenergic signaling. Neuropsychopharmacology.

[CR35] Simms JA, Steensland P, Medina B, Abernathy KE, Chandler LJ, Wise R, Bartlett SE (2008). Intermittent access to 20% ethanol induces high ethanol consumption in Long-Evans and Wistar rats. Alcohol Clin Exp Res.

[CR36] Simms JA, Bito-Onon JJ, Chatterjee S, Bartlett SE (2010). Long-Evans rats acquire operant self-administration of 20% ethanol without sucrose fading. Neuropsychopharmacology.

[CR37] Tolliver GA, Sadeghi KG, Samson HH (1987). Ethanol preference following the sucrose-fading initiation procedure. Alcohol.

[CR38] Vengeliene V, Olevska A, Spanagel R (2015). Long-lasting effect of NMDA receptor antagonist memantine on ethanol-cue association and relapse. J Neurochem.

[CR39] Wise RA (1973). Voluntary ethanol intake in rats following exposure to ethanol on various schedules. Psychopharmacologia.

[CR40] Wouda JA, Diergaarde L, Riga D, Van Mourik Y, Schoffelmeer ANM, De Vries TJ (2010). Disruption of long-term alcohol-related memory reconsolidation: role of β-adrenoceptors and NMDA receptors. Front Behav Neurosci.

