# Supplementary material for: Saccharin fading is not required for the acquisition of alcohol self-administration, and can alter the dynamics of cue-alcohol memory reconsolidation
Source: Psychopharmacology (Berl). 2018 Feb 6;235(4):1069–82. doi: 10.1007/s00213-017-4824-1 (PMC5869889; doi:10.1007/s00213-017-4824-1)
Supplement: Supplementary file 1 — (PDF 328 kb) [file 213_2017_4824_MOESM1_ESM.pdf]

Supplementary Figure 1

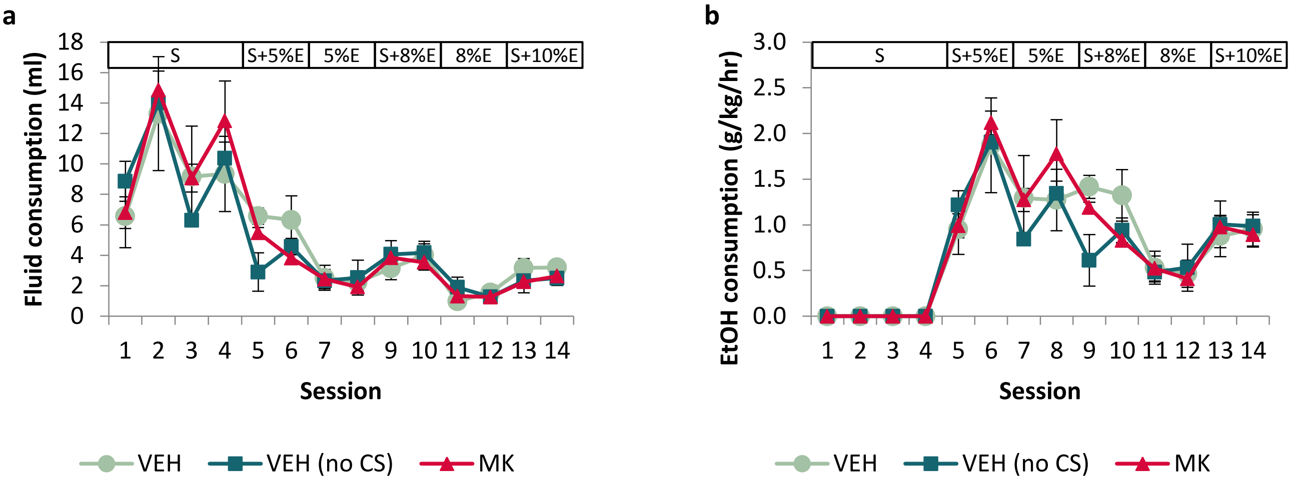

Supplementary Fig. 1 (a) Fluid consumption and **(b)** EtOH consumption during saccharin fading for SF rats. The bar refers to the solution available for each session where S refers to 0.2% saccharin, and E to ethanol of varying concentrations (given in %). Drug groups refer to prospective groupings for subsequent memory reactivation. Group sizes: VEH, n = 4; VEH (no CS), n = 3; MK, n = 5.

Supplementary Figure 2

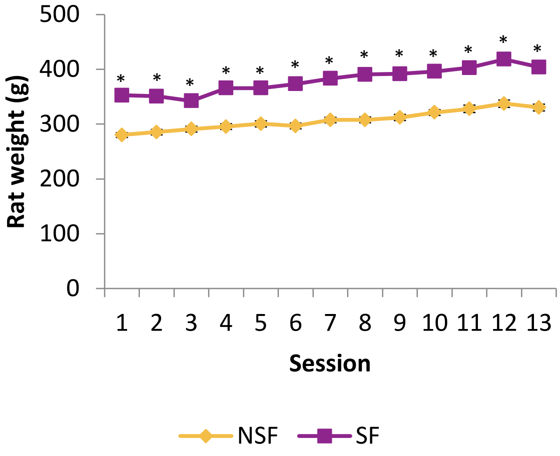

Supplementary Fig. 2 The SF group had a higher average weight than the NSF group across the 13 days of EtOH self-administration training. Group sizes: NSF, n = 12; SF, n = 12. *** $p < 0.05$ comparison of SF to NSF rats.**

Supplementary Figure 3

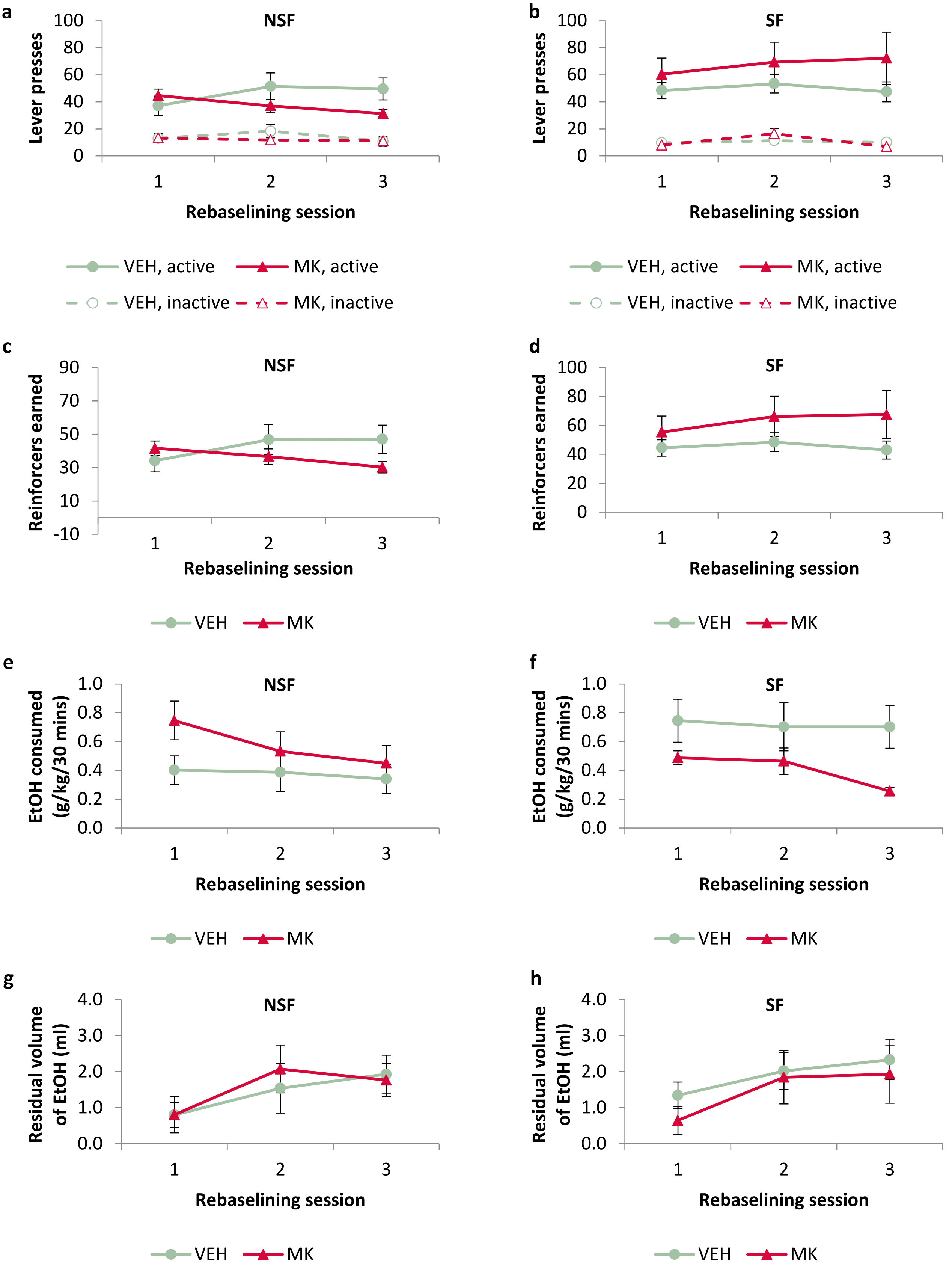

Supplementary Fig. 3 There were no differences between NSF and SF rats, or the different experimental groups , during three 'rebaselining' 10% EtOH self-administration sessions, as measured by **(a, b)** instrumental responding, **(c, d)** the number of reinforcers earned, **(e,f)** the amount of EtOH consumed, or **(g, h)** the volume of 10% EtOH left in the magazine. Group sizes: NSF VEH, n = 7; NSF MK, n = 5; SF VEH, n = 7; SF MK, n = 5.

Supplementary Figure 4

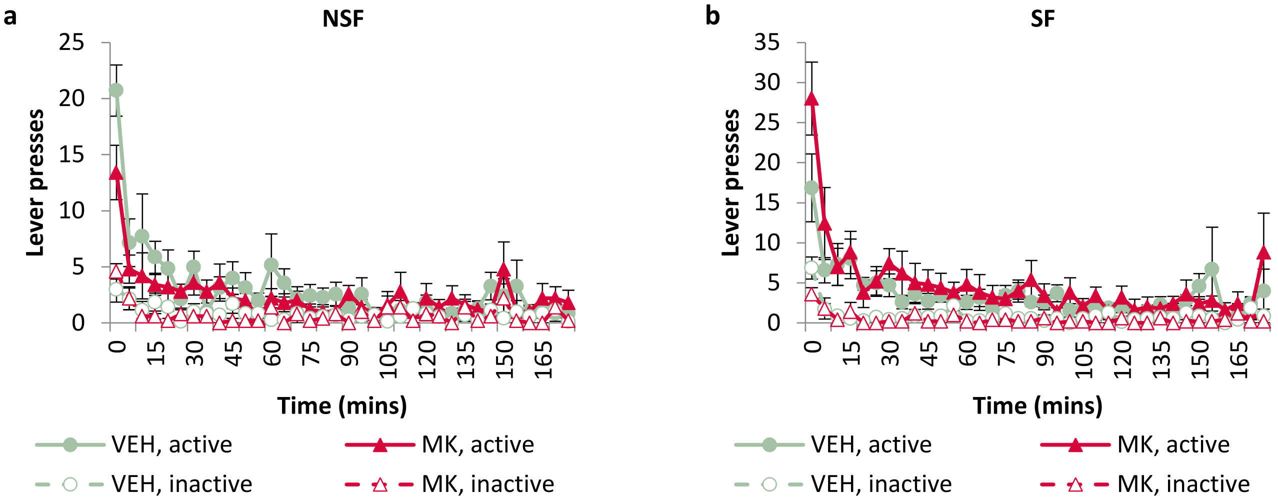

Supplementary Fig. 4 Responding during extinction for **(a)** NSF rats and **(b)** SF rats. Group sizes: NSF VEH, n = 7; NSF MK, n = 5; SF VEH, n = 7; SF MK, n = 5.
